# Cyclodepsipeptides and Other *O*-Containing Heterocyclic Metabolites from *Beauveria felina* EN-135, a Marine-Derived Entomopathogenic Fungus

**DOI:** 10.3390/md12052816

**Published:** 2014-05-13

**Authors:** Feng-Yu Du, Xiao-Ming Li, Peng Zhang, Chun-Shun Li, Bin-Gui Wang

**Affiliations:** 1 College of Chemistry & Pharmacy, Qingdao Agricultural University, Qingdao 266109, China; E-Mail: fooddfy@126.com; 2 Key Laboratory of Experimental Marine Biology, Institute of Oceanology, Chinese Academy of Sciences, Nanhai Road 7, Qingdao 266071, China; E-Mails: lixmqd@aliyun.com (X.-M.L.); zp52715@126.com (P.Z.); lichunshun@ms.qdio.ac.cn (C.-S.L.)

**Keywords:** marine fungus, bryozoan, *Beauveria felina*, secondary metabolites, bioactivity

## Abstract

Bioassay-guided fractionation of a culture extract of *Beauveria felina* EN-135, an entomopathogenic fungus isolated from a marine bryozoan, led to the isolation of a new cyclodepsipeptide, iso-isariin D (**1**); two new *O*-containing heterocyclic compounds that we have named felinones A and B (**2** and **3**); and four known cyclodepsipeptides (**4**–**7**). The structures were elucidated via spectroscopic analysis, and the absolute configurations of **1** and **2** were determined using single-crystal X-ray diffraction and CD, respectively. All isolated compounds were evaluated for antimicrobial activity and brine-shrimp (*Artemia salina*) lethality.

## 1. Introduction

**Figure 1:**
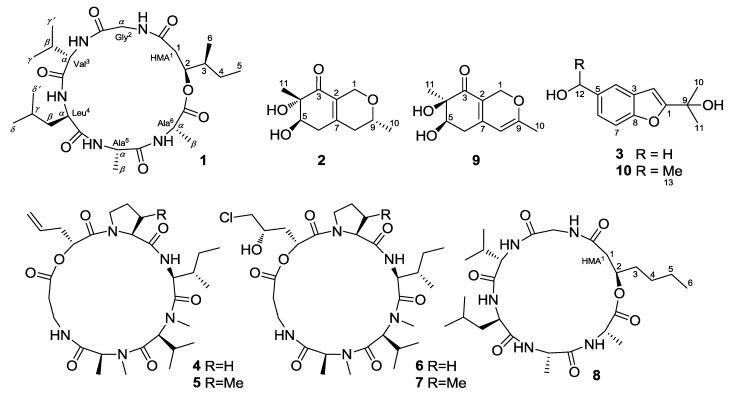
Structures of the isolated compounds **1**–**7** and reference compounds **8**–**10**.

Entomopathogenic fungi such as species from the genera *Beauveria* and *Metarhizium* have been frequently used as an alternative to chemical insecticides for agricultural pest control, and they are attracting increasing attention because of their ability to produce structurally unique and biologically active secondary metabolites [1]. The marine-derived fungal species *Beauveria felina*, which is poorly described, has proven to be a rich source of various cyclodepsipeptides, such as the destruxin, isaridin, and isariin classes, as well as polyketides and terpenoids [2,3,4,5]. As part of our efforts toward the investigation of bioactive secondary metabolites of marine-derived fungi [6,7,8,9,10], *Beauveria felina* EN-135, an entomopathogenic fungus isolated from a marine bryozoan, attracted our attention because of the strong brine-shrimp lethality of the culture extract. Bioassay-guided fractionation of the EtOAc extract led to the isolation and identification of a new cyclodepsipeptide, iso-isariin D (**1**); two new *O*-containing heterocyclic compounds that we have termed felinones A and B (**2** and **3**); and four known cyclodepsipeptides: destruxin A (**4**), roseotoxin B (**5**), destruxin E chlorohydrin (**6**), and [*β*-Me-Pro] destruxin E chlorohydrin (**7**) (Figure 1). The brine-shrimp lethality and antimicrobial activity of these compounds were evaluated. Details of the isolation, structure elucidation, and biological activity of compounds **1**–**7** are reported herein.

## 2. Results and Discussion

### 2.1. Structure Elucidation of the New Compounds

Compound **1** was initially isolated as a colorless amorphous powder. Its molecular formula was established to be C_26_H_45_N_5_O_7_ based on HRESIMS, with seven degrees of unsaturation. Analysis of the ^1^H NMR spectrum of **1** revealed the presence of five amide *NH* protons (*δ*_H_ 9.30, 8.60, 8.54, and two at 8.48), four *N*-CH*α*s (*δ*_H_ 4.84, 4.79, 4.76, and 4.68), one *O*-CH*β* (*δ*_H_ 5.63), one *N*-CH_2_*α* (*δ*_H_ 4.73 and 4.64), and eight methyl groups (*δ*_H_ 1.95, 1.85, 1.55, 1.53, 1.51, 1.49, 1.48, and 1.46). The ^13^C and DEPT NMR spectra of compound **1** exhibited signals of eight methyl groups, four methylenes (one nitrogenated), eight methines (one oxygenated and four nitrogenated), and six ester/amide carbonyl carbons (Table 1). These 1D NMR data indicated that compound **1** is a cyclic hexadepsipeptide with a *β*-hydroxy aliphatic acid moiety [4].

**Table 1:** ^1^H- (500 MHz) and ^13^C-NMR (125 MHz) data for compound 1 in DMSO-*d*_6_ (*δ* in ppm).

Position	*δ*_H_ (*J* in Hz)	*δ*_C_	Position	*δ*_H_ (*J* in Hz)	*δ*_C_
**HMA^1^**	-	-	**Leu^4^**	-	-
CO	-	169.7 C	CO	-	171.1 C
1	3.19, m; 2.92, d (13.6)	37.6 CH_2_	*α*	4.68, m	51.9 CH
2	5.63, m	74.3 CH	*β*	2.13, m	38.6 CH_2_
3	2.20, m	38.0 CH	*γ*	2.28, m	24.1 CH
4	2.03, m; 1.70, m	24.8 CH_2_	*δ*	1.48, d (6.8)	21.1 CH_3_
5	1.49, t (6.9)	11.3 CH_3_	*δ*’	1.53, d (6.8)	18.8 CH_3_
6	1.46, d (6.8)	14.1 CH_3_	NH	9.30, d (6.0)	-
**Gly^2^**	-	-	**Ala^5^**	-	-
CO	-	168.9 C	CO	-	171.6 C
*α*	4.73, m; 4.64, dd (16.4, 3.4)	42.4 CH_2_	*α*	4.84, m	47.5 CH
NH	8.54, br. s	-	*β*	1.85, d (7.0)	17.3 CH_3_
**Val^3^**	-	-	NH	8.60, d (8.3)	-
CO	-	171.7 C	**Ala^6^**	-	-
*α*	4.76, m	58.1 CH	CO	-	171.6 C
*β*	2.51, m	29.9 CH	*α*	4.79, m	48.3 CH
*γ*	1.55, d (7.7)	22.8 CH_3_	*β*	1.95, d (7.2)	16.6 CH_3_
*γ*’	1.51, d (7.7)	18.6 CH_3_	NH	8.48, d (7.6)	-
NH	8.48, d (7.6)	-	-	-	-

**Figure 2:**
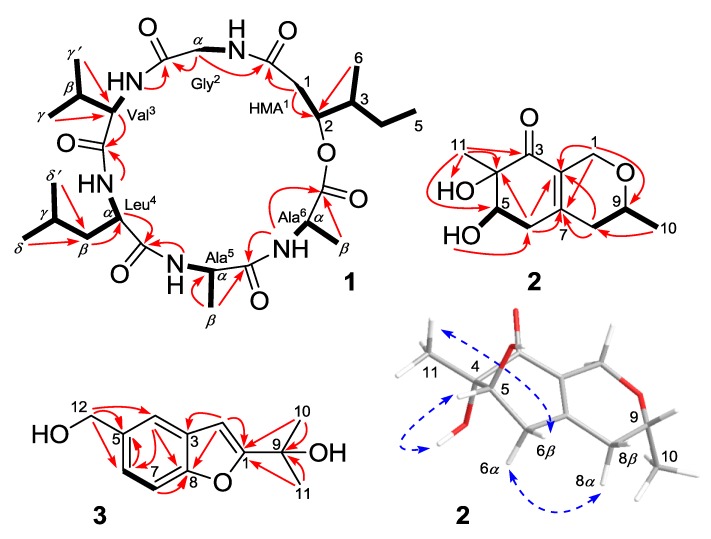
Key ^1^H–^1^H COSY (**bold lines**), HMBC (**red arrows**), and NOE (**dotted blue arrows**) correlations of compounds **1**–**3**.

The assignment of the proton and carbon signals to the amino-acid residues was achieved via COSY, HSQC, and HMBC experiments. Briefly, the five amide *NH* protons were correlated with their corresponding CH*α* protons in the COSY spectrum. Starting from the CH*α* signals, the five amino-acid residues were identified as glycine (Gly^2^), valine (Val^3^), leucine (Leu^4^), and two alanines (Ala^5^ and Ala^6^) via additional COSY and HMBC analysis. The observed HMBC correlations, including CH_2_*α* (Gly^2^) and NH (Val^3^)/CO (Gly^2^), CH*α* (Val^3^) and NH (Leu^4^)/CO (Val^3^), CH*α* (Leu^4^) and NH (Ala^5^)/CO (Leu^4^), *β*-Me (Ala^5^) and NH (Ala^6^)/CO (Ala^5^), and NH (Ala^6^)/CO (Ala^6^), established the amino-acid sequence Gly^2^-Val^3^-Leu^4^-Ala^5^-Ala^6^ (Figure 2). The remainder of the ^1^H and ^13^C NMR signals were unambiguously assigned to a 2-hydroxy-3-methylpentanoic acid moiety (HMA^1^) based on the COSY and HMBC experiments (Figure 2). The HMBC cross-peaks from CH_2_-1 (HMA^1^) and CH_2_*α* (Gly^2^) to CO (HMA^1^) indicated the presence of the hexadepsipeptide sequence *cyclo*(HMA^1^-Gly^2^-Val^3^-Leu^4^-Ala^5^-Ala^6^) in compound **1**, which was consistent with the seven calculated degrees of unsaturation.

**Figure 3:**
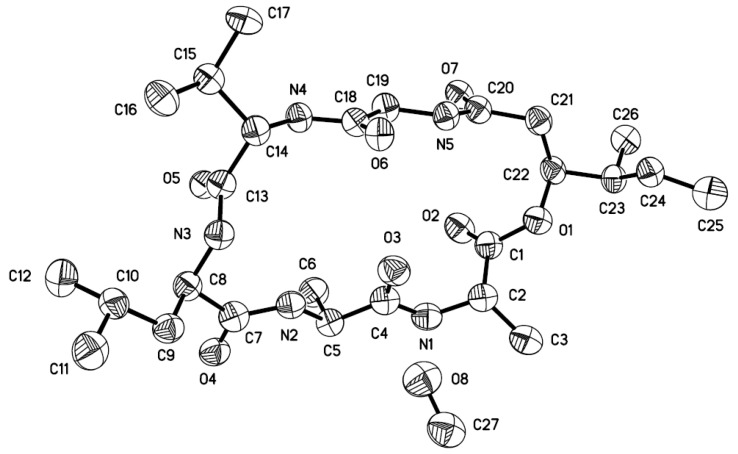
X-ray structure of compound **1** (Note: A different numbering system is used for the structure described in the text).

Compound **1** is considered to be a cyclohexadepsipeptide of the isariin class [11,12,13], and a literature search revealed that the structure of this family of compounds has been primarily determined via spectroscopic analysis and the absolute configuration has been determined by analyzing the amino-acid derivatives, but none of these cyclohexadepsipeptides has been unambiguously determined via X-ray crystallography. We performed crystallization of **1** alongside the spectroscopic studies. Although compound **1** was initially obtained as a colorless amorphous powder, single crystals suitable for X-ray analysis were obtained after many attempts. The structure and absolute configuration of **1** could thus be further determined based on single-crystal X-ray diffraction using Cu K*α* radiation (Figure 3). The result not only confirmed the peptide sequence but also allowed the identification of the amino-acid residues as l-Val^3^, d-Leu^4^, l-Ala^5^, and l-Ala^6^, along with 2*S*-hydroxy-3*S*-methylpentanoic acid (HMA^1^). Most reported cyclodepsipeptides in the isariin class have a 2-hydroxy aliphatic acid moiety characterized with a straight chain [11,12,13]; only one derivative, iso-isariin B, has a branch chain [4]. Compound **1** was the second cyclodepsipeptide of the isariin class to be identified that has this type of unusual HMA moiety, which differentiated it from isariin D (**8**). Thus, the structure of **1** was elucidated and named iso-isariin D. The HMA residue of iso-isariin D (**1**) in *B. felina* is most likely related to the polyketide biosynthesis pathway, and the amino-acid sequence might be obtained from the nonribosomal peptide biosynthesis pathway [14,15].

Compound **2** was obtained as a yellowish solid. The molecular formula C_11_H_16_O_4_ was determined via HRESIMS, implying four degrees of unsaturation (one less than the reference compound **9**) [16]. Detailed analyses of the 1D NMR data (Table 2) indicated the presence of one carbonyl carbon, three additional quaternary carbons (two sp^2^ and one oxygenated sp^3^), two oxygenated sp^3^ methines, three methylenes (one oxygenated sp^3^), and two methyl groups. Comparison between the NMR data of **2** and **9** indicated that their planar structures were very similar except that the signals of the double bond of C-8/C-9 at *δ*_C_ 102.1 (CH) and 165.7 (C) in **9** were replaced by signals of a single bond at *δ*_C_ 36.9 (CH_2_) and 68.6 (CH) in the ^13^C-NMR spectrum of **2**. Accordingly, the proton signal at *δ*_H_ 5.18 (s, H-8) of **9** disappeared in the ^1^H NMR spectrum of **2**. Instead, additional CH_2_ and *O*CH signals at *δ*_H_ 2.07 (m, H-8*α*), 2.20 (m, H-8*β*), and 3.54 (m, H-9) were observed in the ^1^H-NMR spectrum of **2**. The correlations from H-8 to H-9 and from H-9 to H-10 in the COSY spectrum of **2** supported the above deduction (Figure 2).

**Table 2:** ^1^H- (500 MHz) and ^13^C-NMR (125 MHz) data for compounds **2** and **3** (*δ* in ppm).

Compound 2				Compound 3		
Position	*δ*_H_ (*J* in Hz) ^a^	*δ*_H_ (*J* in Hz) ^b^	*δ*_C_^a^	Position	*δ*_H_ (*J* in Hz) ^b^	*δ*_C_^b^
1	4.29, d (15.5) 4.00, d (15.5)	4.43, d (15.8) 4.04, d (15.8)	62.9 CH_2_	1	-	164.1 C
2	-	-	127.3 C	2	6.61, s	100.0 CH
3	-	-	199.1 C	3	-	128.5 C
4	-	-	76.1 C	4	7.52, s	119.2 CH
5	3.74, m	3.91, dd (9.5, 5.3)	71.4 CH	5	-	135.8 C
6*α* 6*β*	2.52, m 2.14, m	2.59, dd (18.1, 5.3) 2.35, dd (18.1, 9.5)	36.7 CH_2_	6	7.24, d (8.4)	123.1 CH
7	-	-	151.6 C	7	7.39, d (8.4)	110.2 CH
8*α* 8*β*	2.07, m 2.20, m	2.12, dd (18.3, 10.1) 2.24, br. s (18.3)	36.9 CH_2_	8	-	154.2 C
9	3.54, m	3.60, ddd (10.1, 6.2, 3.5)	68.6 CH	9	-	68.4 C
10	1.16, d (6.2)	1.21, d (6.2)	20.9 CH_3_	10	1.61, s	27.6 CH_3_
11	1.08, s	1.18, s	18.0 CH_3_	11	1.61, s	27.6 CH_3_
4-*OH*	5.05, br. s	-	-	12	4.65, s	64.1 CH_2_
5-*OH*	5.07, d (4.2)	-	-	-	-	-

^a^ Measured in DMSO-*d*_6_; ^b^ Measured in CD_3_OD.

The relative configuration of compound **2** was determined by analyzing the NOESY data and ^1^H-NMR *J*-values [17]. The observed NOE correlations from *OH*-4 to H-5 and from 11-Me to H-6*β* indicated that they were on the same face of the molecule, respectively (Figure 2). However, the NOEs and coupling constants for H-6 and H-8 were difficult to measure due to overlap with other protons, but a spectrum in CD_3_OD afforded better resolution of these signals. (Table 2). The large coupling constants observed between H-5 and H-6*β* (9.5 Hz) and between H-9 and H-8*α* (10.1 Hz) indicated trans relationships. The key correlation between H-6*α* and H-8*α* in the NOESY spectrum measured in CD_3_OD finally established the relative configuration of **2** as shown in Figure 2.

The absolute configuration of **2** was determined by the application of CD. The CD spectrum displayed a negative Cotton Effect (CE) at 219 nm and a positive CE at 248 nm, which was nearly identical to that of (*S*)-2-acetyl-3,6-dihydroxycyclohex-2-enone, suggesting the presence of an *S*-configuration at C-4, which was opposite to that of pestafolide A [17,18]. The cyclohexenone ring of pestafolide A showed the same relative configuration as that of **2**, but a reverse absolute stereochemistry, which was probably due to a different stereochemistry-selective biosynthetic pathway in *Pestalotiopsis foedan* [17]. The Mosher’s method was also tried to further determine the absolute configuration of **2**, but unsatisfied, probably due to the unstable MTPA esters. Therefore, the absolute configuration of **2** was tentatively assigned to be 4*S*, 5*R*, and 9*R*, and this compound was named felinone A.

Felinone B (**3**) was determined to have the molecular formula C_12_H_14_O_3_ (six degrees of unsaturation) via HRESIMS. The 1D NMR spectra of **3** indicated the presence of four aromatic and one oxygenated sp^3^ quaternary carbons, four sp^2^ methines, one oxygenated sp^3^ methylene, and two methyl groups (Table 2). The structure of **3** was very similar to that of **10**, a compound previously isolated from *Smallanthus* *fruticosus* [19]. The primary difference between compounds **3** and **10** was that the 13-Me and 12-*O*CH proton signals (*δ*_H_ 1.54 and 4.99) of **10** [19] were absent in the ^1^H NMR spectrum of **3** and, instead, an oxygenated methylene signal at *δ*_H_ 4.65 (s, H-12) was observed. Detailed analyses of the COSY and HMBC spectra further confirmed the structure of compound **3** as shown in Figure 2.

In addition to the three novel compounds **1**–**3**, four known cyclodepsipeptides—destruxin A (**4**) [20], roseotoxin B (**5**) [21], destruxin E chlorohydrin (**6**), and [*β*-Me-Pro] destruxin E chlorohydrin (**7**) [3] (Figure 1)—were also isolated and identified from the culture extract of *B.* *felina* EN-135. Their structures were determined via spectroscopic analysis and comparison with previously published reports.

### 2.2. Biological Activities of the Isolated Compounds

The isolated compounds **1**–**7** were evaluated for brine-shrimp lethality and antimicrobial activity. Among them, the hexadepsipeptides **1** and **4**–**7** exhibited potent lethality against brine shrimp (*Artemia salina*), with LD_50_ values of 26.58, 5.34, 0.73, 2.16, and 1.03 μΜ, respectively, which were notably stronger than that of the positive control colchicine (with an LD_50_ value of 88.4 μΜ). Compounds **2** and **3** exhibited weak activity with lethal rates of 61.4% and 59.6%, respectively, at a concentration of 100 μg/mL. The antimicrobial activities against six bacteria (*Escherichia coli*, *Staphylococcus aureus*, *Pseudomonas aeruginosa*, *Vibrio alginolyticus*, *Vibrio anguillarum*, and *Edwardsiella tarda*) and four plant pathogenic fungi (*Physalospora piricola*, *Alternaria brassicae*, *Colletotrichum gloeosporioides*, and *Cucumber fusarium*) were also evaluated. Only compound **3** showed inhibitory activity higher than that of the chloramphenicol control (MIC value of 4 μg/mL) against *P. aeruginosa*; this compound was found to have an MIC value of 32 μg/mL.

## 3. Experimental Section

### 3.1. General

The optical rotations were determined using an Optical Activity AA-55 polarimeter. UV spectra were measured using a Lengguang Gold S54 spectrophotometer (Shanghai Lengguang Technology Co. Ltd., Shanghai, China). The ^1^H, ^13^C, and 2D NMR spectra were acquired using a Bruker Advance 500 spectrometer (Bruker BioSpin Group, Karlsruhe, Germany). Mass spectra were obtained using a VG Autospec 3000 mass spectrometer (VG instruments, London, UK). Semi-preparative HPLC was performed using a Dionex UltiMate U3000 system (Dionex Corporation, Sunnyvale, CA, USA) with an Agilent Prep RP-18 column (21.2 × 250 mm, 10 μm) with UV detection. Column chromatography (CC) was performed using silica gel (200–300 mesh, Qingdao Haiyang Chemical Factory, Qingdao, China), Lobar LiChroprep RP-18 (40–63 μm, Merck, Darmstadt, Germany), and Sephadex LH-20 (18–110 μm, Merck).

### 3.2. Fungal Material

The fungus *Beauveria felina* EN-135 was isolated from an unidentified marine bryozoan and identified via sequence analysis of the ITS region of its rDNA as previously described [22]. The sequence data derived from the fungus, which was similar (99%) to the sequence of *Beauveria felina* CBS 250.34 (compared to accession No. AY261369), was deposited in GenBank with the accession No. HQ891664. The strain was preserved at the Key Laboratory of Experimental Marine Biology at the Institute of Oceanology of the Chinese Academy of Sciences.

### 3.3. Fermentation

The fungal strain was statically fermented at r.t. for 40 days on rice solid medium containing rice (100 g/flask), peptone (0.6 g/flask), and sea water (100 mL/flask) in 1 L Erlenmeyer flasks (×60).

### 3.4. Extraction and Isolation

The fermented rice medium was exhaustively extracted using EtOAc to obtain a crude extract (25.1 g), which was subjected to silica-gel vacuum liquid chromatography (VLC) and eluted with mixed solvents of increasing polarity (petroleum ether-EtOAc, 20:1 to 1:1, followed by CHCl_3_-MeOH, 40:1 to 1:1) to yield nine fractions (Frs. 1–9). Frs. 7 and 8 exhibited potent lethality against brine shrimp, with LD_50_ values of 17.27 and 11.67 μg/mL, respectively. Fr. 7 (3.4 g) was separated into five subfractions (Frs. 7. 1–5) via CC on RP-18 (MeOH/H_2_O, from 1:9 to 1:0). Fr. 7.1 (179.2 mg) was subjected to CC on Sephadex LH-20 (MeOH) and was further purified via semi-preparative HPLC (30% MeOH/H_2_O, 16 mL/min) to yield compounds **2** (*t*_R_ = 11.6 min, 5.1 mg) and **3** (*t*_R_ = 16.3 min, 18.6 mg). Fr. 7.3 (429.4 mg) was subjected to CC on silica gel eluted with CHCl_3_-MeOH (100:1 to 20:1) and Sephadex LH-20 (acetone) and was further purified via semi-preparative HPLC (40% MeCN/H_2_O, 16 mL/min) to yield compounds **4** (*t*_R_ = 12.8 min, 16.0 mg) and **5** (*t*_R_ = 15.8 min, 15.2 mg). Fr. 8 (2.3 g) was separated via CC on RP-18 (MeOH/H_2_O, from 1:9 to 1:0) to yield four subfractions (Frs. 8. 1–4). Fr. 8.2 (387.6 mg) was subjected to CC over silica gel eluted with CHCl_3_-MeOH (50:1 to 10:1) and was purified via semi-preparative HPLC (65% MeOH/H_2_O, 16 mL/min) to yield compounds **6** (*t*_R_ 13.5 min, 39.1 mg) and **7** (*t*_R_ 17.3 min, 19.5 mg). Fr. 8.3 (108.7 mg) was separated via CC on Sephadex LH-20 (acetone) and then via semi-preparative HPLC (45% MeCN/H_2_O, 16 mL/min) to yield compound **1** (*t*_R_ 12.8 min, 10.4 mg).

Iso-isariin D (**1**): colorless crystal; mp 258–259 °C; 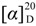: −22.2 (*c* 0.36, MeOH); UV (MeOH) *λ*_max_ (log *ε*) 206 (3.64) nm; ^1^H and ^13^C NMR data, see Table 1; ESIMS *m*/*z* 540 [M + H]^+^; HRESIMS *m*/*z* 540.3392 [M + H]^+^ (calcd for C_2__6_H_46_N_5_O_7_, 540.3392).

Felinone A (**2**): yellowish solid; 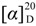: +104.8 (*c* 0.21, MeOH); UV (MeOH) *λ*_max_ (log *ε*) 200 (3.45), 240 (3.77) nm; CD *ë*_max_ (*Δε*) 219 (−13.89), 248 (+27.06) nm; ^1^H and ^13^C NMR data, see Table 2; ESIMS *m*/*z* 213 [M + H]^+^; HRESIMS *m*/*z* 213.1123 [M + H]^+^ (calcd for C_11_H_17_O_4_, 213.1121).

Felinone B (**3**): yellowish solid; 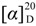: −16.7 (*c* 0.12, MeOH); UV (MeOH) *λ*_max_ (log *ε*) 209 (4.38), 247 (4.09), 279 (3.47), 286 (3.48) nm; ^1^H and ^13^C NMR data, see Table 2; ESIMS *m*/*z* 229 [M + Na]^+^; HRESIMS *m*/*z* 229.0828 [M + Na]^+^ (calcd for C_12_H_14_O_3_Na, 229.0835).

### 3.5. X-ray Crystallographic Analysis of Compound 1

The crystallographic data were collected using a Bruker D8-advance X-ray diffractometer (Bruker AXS Corporation, Karlsruhe, Germany) equipped with graphite monochromatic Cu-*Kα* radiation (*λ* = 1.54178 Å) at 293(2) K [23]. The absorption data were obtained using the program SADABS [24]. The structure was analyzed via direct methods using the SHELXTL software package [25]. All non-hydrogen atoms were refined anisotropically. The H atoms were located via geometrical calculations, and their positions and thermal parameters were fixed during structure refinement. The structure was refined using full-matrix least-squares techniques [26].

Crystal data for compound **1**: C_2__6_H_45_N_5_O_7_·CH_3_OH, F.W. = 571.71, one molecule containing a CH_3_OH solvent molecule in the unit, orthorhombic, space group P2(1)2(1)2(1), unit-cell dimensions *a* = 9.5437(7) Å, *b* = 15.5388(13) Å, *c* = 22.315(2) Å, *α* = *β* = *γ* = 90°, *V* = 3309.3(5) Å^3^, *Z* = 4, *d*_calcd_ = 1.147 mg/m^3^, crystal dimensions 0.24 × 0.10 × 0.06 mm^3^, *μ* = 0.696 mm^−^^1^, *F*(000) = 1240. The 17936 measurements yielded 5791 independent reflections after equivalent data were averaged, and Lorentz and polarization corrections were applied. The final refinement yielded *R*_1_ = 0.1104 and w*R*_2_ = 0.1978[*I* > 2*σ*(*I*)]. The Flack parameter was 0.0 (13) in the final refinement for all 5791 reflections with 372 Friedel pairs.

### 3.6. Brine-Shrimp Lethality

The brine-shrimp (*Artemia salina*) lethality of the isolated compounds was determined as previously described [27]. Colchicine was used as a positive control.

### 3.7. Antimicrobial Assay

The antibacterial activities against *E. coli*, *S. aureus*, *P. aeruginosa*, *V. alginolyticus*, *V. anguillarum*, and *E. tarda* along with the antifungal activities against *P. piricola*, *A. brassicae*, *C. gloeosporioides*, and *C. fusarium* were investigated using the disk diffusion and double dilution methods as previously described [28,29]. Chloramphenicol and amphotericin B were used as positive controls for the antibacterial and antifungal bioassays, respectively.

## 4. Conclusions

One novel cyclodepsipeptide, iso-isariin D (**1**), and two novel *O*-containing heterocyclic compounds, felinones A and B (**2** and **3**), were isolated from a culture of *Beauveria felina* EN-135. In addition, four known destruxin cyclodepsipeptides (**4**–**7**) were also identified. The structures were elucidated via spectroscopic analysis, and the absolute configurations of **1** and **2** were determined using single-crystal X-ray diffraction and CD, respectively. Compounds **1** and **4**–**7** exhibited potent brine-shrimp lethality with LD_50_ values of 26.58, 5.34, 0.73, 2.16, and 1.03 μΜ, respectively, whereas compound **3** showed inhibitory activity against *P. aeruginosa* with an MIC value of 32 μg/mL.
